# An Oligopeptide Transporter of *Mycobacterium tuberculosis* Regulates Cytokine Release and Apoptosis of Infected Macrophages

**DOI:** 10.1371/journal.pone.0012225

**Published:** 2010-08-17

**Authors:** Arunava Dasgupta, Kamakshi Sureka, Devrani Mitra, Baisakhee Saha, Sourav Sanyal, Amit K. Das, Parul Chakrabarti, Mary Jackson, Brigitte Gicquel, Manikuntala Kundu, Joyoti Basu

**Affiliations:** 1 Unité de Génétique Mycobactérienne, Institut Pasteur, Paris, France; 2 Department of Chemistry, Bose Institute, Kolkata, India; 3 Department of Biotechnology, Indian Institute of Technology, Kharagpur, India; University of Delhi, India

## Abstract

**Background:**

The *Mycobacterium tuberculosis* genome encodes two peptide transporters encoded by Rv3665c-Rv3662c and Rv1280c-Rv1283c. Both belong to the family of ABC transporters containing two nucleotide-binding subunits, two integral membrane proteins and one substrate-binding polypeptide. However, little is known about their functions in *M. tuberculosis*. Here we report functional characterization of the Rv1280c-Rv1283c-encoded transporter and its substrate-binding polypeptide OppA_MTB_.

**Methodology/Principal Findings:**

OppA_MTB_ was capable of binding the tripeptide glutathione and the nonapeptide bradykinin, indicative of a somewhat broad substrate specificity. Amino acid residues G109, N110, N230, D494 and F496, situated at the interface between domains I and III of OppA, were required for optimal peptide binding. Complementaton of an *oppA* knockout mutant of *M. smegmatis* with OppA_MTB_ confirmed the role of this transporter in importing glutathione and the importance of the aforesaid amino acid residues in peptide transport. Interestingly, this transporter regulated the ability of *M*. *tuberculosis* to lower glutathione levels in infected compared to uninfected macrophages. This ability was partly offset by inactivation of *oppD*. Concomitantly, inactivation of *oppD* was associated with lowered levels of methyl glyoxal in infected macrophages and reduced apoptosis-inducing ability of the mutant. The ability to induce the production of the cytokines IL-1β, IL-6 and TNF-α was also compromised after inactivation of *oppD*.

**Conclusions:**

Taken together, these studies uncover the novel observations that this peptide transporter modulates the innate immune response of macrophages infected with *M. tuberculosis*.

## Introduction

ATP binding cassette (ABC) transporters are found both in eukaryotes and prokaryotes, and play important roles in the transport of ions, amino acids, peptides, proteins, etc. [1]. ABC transporters may be importers or exporters depending on the direction in which they transport substrates [2]. Importers are found exclusively in prokaryotes and include the oligopeptide transporter systems [3]–[6].

The oligopeptide importers consist of five subunits: two homologous integral membrane proteins OppB and OppC which form the translocation pore, two nucleotide-binding domains OppD and OppF and the substrate-binding lipoprotein (SBP) OppA that determines substrate specificity. In Gram-positive bacteria SBPs are soluble proteins residing in the periplasm. They are either anchored to the membrane through a lipid modification on the N-terminal cysteine or covalently linked to the translocation pore [7]. Crystal structures are available for the dipeptide-binding protein DppA from *E. coli* [8], OppA from *Salmonella typhimurium* [9], OppA from *Yersinia pestis* [10], AppA from *Bacillus subtilis* [11] and OppA from *Lactococcus lactis* [12]. The structures explain the promiscuity of the proteins for peptide binding. The binding pockets are fairly large. Substrate binding is determined by hydrogen bonds with the substrate backbone. In the case of the *L. lactis* OppA, the binding cavity is exceptionally large.

The genome sequence of *M. tuberculosis* H37Rv reveals two peptide permease operons encoded by *Rv3665c-Rv3662c* and *Rv1280c-1283c* [13]. Disruption of the homolog of the Rv1280c to 1283c-encoded peptide transporter in *M. bovis* BCG renders the resulting mutant resistant to the toxic peptides glutathione and S-nitrosoglutathione [13]. However, in their study, the authors did not analyze the substrate-binding properties of the binding protein, OppA in particular. On the other hand, broad substrate specificity has been demonstrated for the three OppA proteins of *Borrelia burgdorferi* [14].

In order to understand the biological roles of the putative peptide importers of mycobacteria, we have cloned, expressed and studied the substrate-binding properties of OppA of *M. tuberculosis* (OppA_MTB_). We observe that OppA is capable of binding both the tripeptide glutathione and the nonapeptide bradykinin. Based on homology modeling and mutational analysis, we have identified amino acid residues that are critical for substrate binding. Further, we have used an *oppA*-knock out mutant (OppA-KO) of *M. smegmatis* as a model system to demonstrate that the Opp transporter is capable of importing glutathione. This observation provided the motivation to test the possible role of this transporter in *M. tuberculosis* that has infected macrophages. The detoxification of reactive ketoaldehydes such as methylglyoxal (MG) by glyoxalase I and II protects cells from formation of advanced glycation end products (AGEs). Glutathione is a cofactor in these reactions [15], [16]. We contended that the import of glutathione by bacilli in infected macrophages, could possibly affect the ability of the macrophages to convert MG to lactate.

We present evidence that MG levels are lower in macrophages infected with an *oppD* knock out (OppD-KO) of *M. tuberculosis* compared to the wild type. In keeping with this, we observed decreased apoptosis of macrophages infected with OppD-KO compared with the wild type. Knock out of *oppD* also altered the ability of the bacterium to trigger cytokine release from infected macrophages. The release of TNF-α, IL-6 and IL-1β was attenuated in the mutant compared to the wild type.

## Materials and Methods

### Molecular biological procedures

Standard procedures were used for cloning and analysis of DNA, PCR and transformation. Electroporation in mycobacteria was carried out using a Bio-Rad Gene Pulser as described by Snapper *et al*. [17]. Enzymes used to manipulate DNA were from Roche Applied Sciences. All constructs made by PCR were sequenced to verify their integrity.

### Bacterial strains and growth conditions

*Escherichia coli* strains were grown in Luria–Bertani (LB) Miller (Difco) medium. Mycobacterial strains were grown in Middlebrook (MB) 7H9 (Difco) supplemented with 2% glucose, 0.05% Tween 80 or Lemco medium supplemented with 0.05% Tween 80. Antibiotics were used at the following concentrations: ampicillin, 75 µg/ml; kanamycin monosulfate, 50 µg/ml for *E. coli* and 25 µg/ml for *M. smegmatis*; and hygromycin B, 100 µg/ml for *E. coli* and 50 µg/ml for *M. smegmatis.*

### Construction of expression plasmids for OppA

*oppA* (Rv1280c) of *M. tuberculosis* was amplified from cosmid MTCY50 using the primer pair 5′-TTT CTA GAC ATA TGG CTG ACC GTG GCC AG-3′ (sense) and 5′- TCA GCG TCG CAT GAA CCC GAT GGC-3′ (antisense) and cloned into the vector pK19 digested with SmaI to generate pOpp101. The resulting construct was digested with NdeI and HindIII and the excised fragment was cloned between the same sites of pET28a^+^ (Novagen) to generate pOpp102. Mutants of *oppA* were generated by overlap extension PCR. The primers used are given in Table S1 with restriction sites in bold. The initial rounds of PCR were carried out using primer pairs “a” and “b”, and “c” and “d” and pOpp102 as template. The products of each PCR were purified and used as templates for the second round of PCR using primers “a” and “d”. The final products were cloned between the NheI and EcoR1 sites of pET28a^+^ to generate mutants of OppA of *M. tuberculosis* in pET 28a^+^. The integrity of all constructs was checked by sequencing.

### Expression and purification of OppA

Recombinant plasmids derived from pET 28a^+^ were transformed in *E. coli* BL21(DE3). Cells were grown to an OD_600_ of 0.6. IPTG was added to a final concentration of 250 µM and growth was continued at 37°C with shaking for 2 h. Cells were harvested and resuspended in 10 mM Tris-HCl (pH 7.4), 1 mM MgCl_2_, 1 mM PMSF, 20 µg/ml leupeptin, 10 µg/ml pepstatin and 10 µg/ml aprotinin, and disrupted by sonication. Recombinant His-tagged proteins were purified from lysates by chromatography on Ni^2+^-NTA agarose.

### In vitro binding assays

Purified OppA (1 µg) was added to a 25 µl reaction volume containing the binding buffer (25 mM Na-phosphate (pH-6.5), 100 mM NaCl). 0.1 M DTT was added when using glutathione as a substrate. The reaction was started by addition of 3,4(n)-^3^H bradykinin (specific activity 7 Ci/mmol, GE Healthcare), or glutathione (specific activity 52 Ci/mmol, Perkin Elmer) at various concentrations and continued at 25°C for 15 min. The reaction mix was then subjected to TCA precipitation, the precipitate was dried and counted in a liquid scintillation counter.

### Uptake of [^3^H] GSH

*M. smegmatis* cells were grown up to an OD_600_ of 0.6, washed in basal salts containing 0.05% Tween 80, and concentrated to an OD_600_ of 3.0. The cell suspension (1 ml) was warmed to 37°C with shaking. The uptake reaction was initiated by the addition of radiolabeled substrate along with unlabeled substrate at a specific activity of 4.48 mCi/mmol and a final concentration of 100 mM. Incorporation was terminated by removal of 0.1-ml samples at the indicated time points, and filtration on Whatman GF/C (0.45-µm pore size) filters prewetted with basal salts. The cells were quickly washed thrice with 5 ml of ice-cold basal salts containing Tween 80 on a vacuum filtration manifold. Filters were counted in a liquid scintillation counter. Uptake assays were performed in triplicate.

### Molecular modeling

Using the BLAST server (http://www.ncbi.nlm.nih.gov/BLAST/), template structures homologous to OppA were sought, but no appreciable homology was found in the Brookhaven pdb database. Appropriate template structures were therefore chosen by comparing the results of fold prediction servers 3DPSSM (http://www.sbg.bio.ic.ac.uk/~3dpssm/), GENTHREADER (http://bioinf.cs.ucl.ac.uk/psiform.html), FFAS (http://www. bioinformatics.burnham-inst.org/FFAS/index.html), UCLA-DOE (http://fold.doe-mbi.ucla.edu/psiform/), BIOINBGU (http://www.cs.bgu.ac.il/~bioinbgu/), and FUGUE (http://www-cryst.bioc.cam.ac.uk/~fugue/). The best template which came out from these analyses was OppA of *Salmonella typhimurium* complexed with the peptide KAK (pdb code 1JET). This was used as template to model the structure of OppA of *M. tuberculosis* (OppA_MTB_) using the software MODELER. The output was a 3D model for the target sequence containing all main chain and side chain non-hydrogen atoms. Amino acid residues I77 to R591 of OppA_MTB_ were modeled. The N-terminal 76 residues could not be modeled due to lack of homology. A total of 20 models were generated and the final model was selected based on its stereochemical properties. This model was subjected to energy minimization using the Insight II Version 2000 (Accelrys Inc.) software package, using the steepest descent minimization algorithm in the discover module of Insight II. The overall stereochemical quality of the final models was assessed by the program PROCHECK. The resolution was set at 1.5 Å. The final models were verified through the program VERIFY 3D (http://www.doe-mbi.ucla.edu/Services/Verify_3D/) and ERRAT (http://www.doe-mbi.ucla.edu/Services/Errat.html).

### Docking of OppA with glutathione and bradykinin

The co-ordinates of bradykinin were obtained from PRODRG server and the docking was performed with AUTODOCK 3.0.5. For docking of OppA with glutathione, the glutathione coordinates were taken from glutathione-bound human glutathione-S transferase structure (PDB ID: 1AQW) [18]. Bradykinin was drawn with JME Molecular Editor using the PRODRG server (http://davapc1.bioch.dundee.ac.uk/cgi-bin/prodrg_beta) and the co-ordinate file was generated. All hydrogen atoms in both protein and ligands were explicitly modeled, with polar hydrogen atoms being assigned Lennard-Jones 12-10 hydrogen bonding parameters and nonpolar hydrogen atoms being assigned 12-6 parameters. Hydrogen atoms were added to the OppA structure using the builder module of the Insight II software package. All water molecules were removed while docking. Partial charges were assigned to the protein atoms using CVFF force field of Insight II. Atomic solvation parameters and fragmental atom volumes were added using the AddSol program provided in the Autodock 3.0.5 suite. The grid maps for van der Waals and electrostatic energies were prepared using AutoGrid version 3.0 with 50×44×40 points spaced at 0.375 distances. The active sites of several OppA crystal structures were screened first and these were found to be similar. Hence the grid was centered in the active site accordingly. The main aim of this docking study was to identify the active site residues by determining the bound conformation of glutathione/bradykinin in the active site of OppA and correlating the result with mutational analysis. All docking jobs were run on an SGI O2 with R5000 processor running IRIX 6.5.

### Construction of suicidal delivery vector for inactivation of *oppA* of *M. smegmatis*

*M. smegmatis* mc^2^ 155 *oppA*, -*B*, -*C* and –*D* are encoded by the ORFs *MSMEG_4999*, *MSMEG_4995*, *MSMEG_4996* and *MSMEG_4997* respectively. The unmarked deletion mutant of *oppA* of *M. smegmatis* was constructed using the allelic replacement method of Parish and Stoker [19]. Briefly, the *oppA* gene along with its upstream sequence was PCR amplified in two steps to create an in-frame deletion, from the genomic DNA of the wild type. Fragment 1 was amplified with the primer pair 5′-TAT AAG CTT CGA CAA GTC CGC GCG CTC-3′ (sense) and 5′-TAA TTC TAG AAC GGC TCG GCG AAC GTC A -3′ (antisense) and fragment 2 was amplified using the primer pair 5′-ATA TTC TAG AAG GCC AGG ACG ATG GCC AA -3′ (sense) and 5′- TAG GAT CCT CGA CGA TGC GGG CGT CGG CA -3′ (antisense) [restriction sites underlined]. Fragment 1 (1060 bp) was cloned between the Hind III and XbaI sites of the vector pUC19 to generate pOpp201. Fragment 2 (1064 bp) was cloned between the XbaI and BamHI sites of pOpp201 to generate pOPP202. The 2.1 kb insert carrying the disrupted *oppA* gene (with a deletion of 988 bp) was excised with HindIII and BamHI and cloned between the same sites of p2NIL to generate pOpp203. The final delivery vector pOpp204 was generated by cloning the PacI cassette (hyg, p*Ag85*- *lac Z*, p*hsp60*- *sac* B) excised from the vector pGOAL 19 into the PacI site of pOPP203.

### Isolation of the mutant inactivated in the *oppA* gene

Denatured pOpp204 DNA was electroporated into electrocompetent cells of *M. smegmatis*. The cells after electroporation were revived in 5 ml of Lemco media for 3 h and then plated on Lemco agar supplemented with hygromycin B, kanamycin and 50 µg/ml X-gal. Blue colonies appeared after 3 days. These were replated onto Lemco agar without any selection to enhance the recombination process. From the colonies that appeared on the plates, a loopful of cells was taken and resuspended in Lemco broth, mixed with vortexing, serial dilutions were plated onto Lemco agar supplemented with 2% sucrose and 50 µg/ml X-gal and allowed to grow for two days. The white colonies that appeared after 2 days on the plates were then streaked onto 4 replica plates, the first supplemented with X-gal, the second with kanamycin, the third with hygromycin B and the fourth with sucrose. The white, kanamycin and hygromycin-sensitive, sucrose-resistant colonies were identified, analyzed by PCR and candidate *opp A* mutants were confirmed by Southern analysis.

### Complementation of *oppA*_MTB_ in the OppA-KO

Complementation of *oppA* was achieved by constructing an integrating vector containing a hygromycin-resistance cassette along with a positive-selection L5 integrase cassette. *oppA* of *M. tuberculosis* (wild type or variants) was first cloned in pOLYG [20], under the control of the *hsp60* promoter and *hsp60–oppA* was excised and cloned between the same sites in pUC19 to generate pOPP205. A 3.7 kb Hyg-integrase cassette from pUC-HY-INT [21], was cloned at the single HindIII site of pOpp205 to generate the integrating vector pOpp206. pOpp206 was electroporated into electrocompetent cells of the OppA-KO strain in order to complement the mutant strain with a single copy of the wild type *oppA* of *M. tuberculosis*. The presence of the *oppA*_MTB_ gene was confirmed by PCR with *oppA-*specific primers. Variants of *oppA*_MTB_ were similarly complemented in OppA-KO.

### Construction of the *M. tuberculosis oppD* knock out mutant

The *M. tuberculosis oppD* mutant was constructed by targeted mutagenesis using a temperature sensitive-*sacB* delivery system as described previously [22]. Amplification of the *oppD* gene along with flanking regions was done in two steps to delete the conserved functional sites. In the first step a 948 bp fragment of *oppD* along with its upstream region was PCR amplified using the forward and reverse primers 5′- TAT ATC TAG *A*AC CGT TGA TCG CCA ACG G -3′ and reverse 5′- TAG AAT TCG AGA ACG GCT CGG CGA A -3′ respectively and cloned in pBluescript SK (Stratagene) between the XbaI and EcoRI sites (underlined) to generate pOppD101. In the second step, a 1037 bp fragment encoding the C-terminus of OppD along with the downstream flanking region was amplified using the forward and reverse primers 5′-AT*G AAT TC*G GCA GGA TCA GCA GCC CGG A-3′ and 5′-TA*A AGC TT*C TAG ATG CGC ACC GCC ATC-3′ respectively and cloned in pOppD101 between the EcoRI and HindIII sites (italicized) to generate pOppD102. pOppD102 was digested with XbaI and ligated with a kanamycin resistance gene excised from pUC4K to generate pOppD103. A 3.2 kb fragment containing the disrupted *oppD* along with the kanamycin resistance cassette was digested with XbaI and cloned into pPR27*xylE* which harbors the *sacB* and *xylE* genes [22] to generate pOppD104. The resulting vector, pOppD104 was electroporated into *M. tuberculosis* H37Rv and transformants were selected at 32°C on kanamycin plates. Single colonies were grown in broth at 32°C and then plated on sucrose plates at 39°C. The resulting colonies were analyzed by PCR and candidate *oppD* mutants were confirmed by Southern analysis.

### Complementation of *oppDA* of *M. tuberculosis* in the OppD-KO

Since the *oppA* gene is downstream of *oppD*, its expression was blocked in the OppD-KO. Complementation was therefore carried out with *oppDA* by constructing an integrating vector containing a hygromycin-resistance cassette along with a positive-selection L5 integrase cassette. The *oppDA* coding region of *M. tuberculosis* was PCR amplified in two fragments. The first fragment was amplified with the primer pair 5′-TAG AAT TCA TAT GAG CCC CCT GCT CGA-3′ (sense) and 5′-GGG GCG CAG CCT CGT CGC TGG CCA-3′ (antisense) and cloned between the sites EcoRI and SmaI sites of the vector pUC19 to generate the construct pOppD105. The second fragment was amplified with the primer pair 5′-GGG CAT CGC GTC TGC GCT CAG GGC GTC-3′ (sense) and 5′-TAT AAG CTT TCA GCG TCG CAT GAC CCC-3′ (antisense) and cloned between the SmaI and HindIII sites of pOppD105. The resulting construct pOppD106 was digested with NdeI and HindIII and the excised fragment containing the *oppDA* region was cloned under the *hsp* promoter of an *E. coli*- mycobacteria shuttle vector digested with the same enzymes to generate the construct pOppD107. *hsp60–oppDA* was excised with XbaI and HindIII and cloned between the same sites in pUC19 to generate pOppD108. The 3.7 kb Hyg-integrase cassette from pUC-HY-INT was cloned at the single HindIII site of pOppD108 to generate the integrating vector pOppD109. pOppD109 was electroporated into electrocompetent cells of the knockout strain in order to complement the mutant strain with a single copy of the wild type *oppDA* of *M. tuberculosis*. The presence of the *oppDA* gene was confirmed by PCR with *oppDA-*specific primers.

### Cell death ELISA

Differentiated THP-1 cells were plated in 96 well plates (10^5^ cells/well). Wild type *M. tuberculosis* H37Rv or OppD-KO was grown in MB 7H9 medium supplemented with ADC and 0.05% Tween 80 for 2 days for getting a well dispersed culture having O.D._600_ value between 0.2 and 0.4. Infection at the indicated MOI was done for 2 h; cells were washed twice with 1X RPMI to remove the unphagocytosed bacteria. For determination of CFUs, cells were solubilized in 0.06% SDS, lysates were serially diluted and plated. At the MOIs used, there was a linear relationship between the MOI and the actual number of bacteria infecting macrophages. Fresh medium was added to each well and medium was changed every day. Finally, cells were lysed with lysis buffer supplied with the cell death ELISA kit (Roche Applied Science). ELISA was performed according to the manufacturer's protocol.

### Determination of methylglyoxal (MG) Levels in THP-1 cells

Measurement of MG levels was performed as described by Rachman *et al*. [23] with some modifications. THP-1 cells were infected with wild type *M. tuberculosis* or the *oppD* knockout (OppD-KO) at an MOI of 20:1 for 2 h. One day after infection, THP-1 cells were scraped, harvested, washed twice with PBS, and lysed by sonication in PBS. Perchloric acid (PCA) (Merck) and *o*-phenylenediamine (Across) were added to a final concentration of 0.5 M and 5 mM, respectively. The mixture was incubated at 20°C for 24 h. The PCA precipitate was removed by centrifugation at 12,000×*g*. The supernatant was passed through a C18 solid phase extraction cartridge (Waters Sep-Pak C18 plus cartridge, Millipore), which had been flushed with 6–8 ml of acetonitrile and 6–8 ml of 10 mM KH_2_PO_4_ (pH 2.5). The sample was eluted from the cartridge with 4 ml acetonitrile (Sigma). The quinoxaline derivative of methylglyoxal (2-MQ) and the quinoxaline internal standard (5-MQ) (Across) were measured using an Adsorbosphere 25 cm C-18 column (4.6 mm internal diameter and 5 µm particle diameter) on a Waters chromatography system. The mobile phase was 68 vol% of 10 mM KH_2_PO_4_ (pH 2.5) and 32 vol% of acetonitrile. The analysis conditions were as follows: detector wavelength, 315 nm; mobile phase flow rate, 1.0 ml/min; typical sample size, 150 µl; and column temperature, 20°C. Duplicate injections of each sample were made. Samples were calibrated with a 2-MQ internal standard. The average retention times of 2-MQ and 5-MQ were 6.3 and 8.5 min, respectively. Increase in MG was determined by calculating the ratio of peak areas between cells with and without infection. The ratio was normalized with the ratio of the corresponding cell numbers.

### Measurement of intracellular glutathione level

Glutathione was measured using the Glutathione Fluorimetric Detection Kit (Abcam, UK) according to the manufacturer's protocol. Briefly, 10^6^ differentiated THP-1 cells were infected and lysed by treating with 100 µl of ice cold lysis buffer. The cell lysate was diluted and mixed with GST reagent and MCB dye (supplied by the manufacturer). After an incubation of 30 min at 37°C, fluorescence was measured with excitation at 380 nm and emission at 460 nm.

## Results

### Binding specificity of OppA

The annotated *M. tuberculosis* genome indicates the presence of two peptide permeases *opp* (*Rv1280c-Rv1283c*) [*Rv1280c* (*oppA*), *Rv1281c* (*oppD*), *Rv1282c* (*oppC*), *Rv1283c* (*oppB*)and *dpp* (*Rv3665c-Rv3662c*). Recent studies on *M. bovis* BCG suggest that the *opp* operon encodes an oligopeptide transporter [24]. On the other hand, Flores-Valdez *et al*. [25], have argued that *Rv3665c-Rv3662c* encodes the oligopeptide transporter of *M. tuberculosis*, and that the *Rv1280c-Rv1283c* operon likely encodes a dipeptide transporter. Their arguments rely on the fact that an *Rv3665c-Rv3662c* knockout mutant is resistant to bialaphos. However, no binding studies for the substrate-binding components of these transporters have been reported by this group. We undertook a more detailed analysis of the *Rv1280c-Rv1283c* operon. As a first step, we attempted to characterize OppA in terms of its binding specificity.

**Figure 1:**
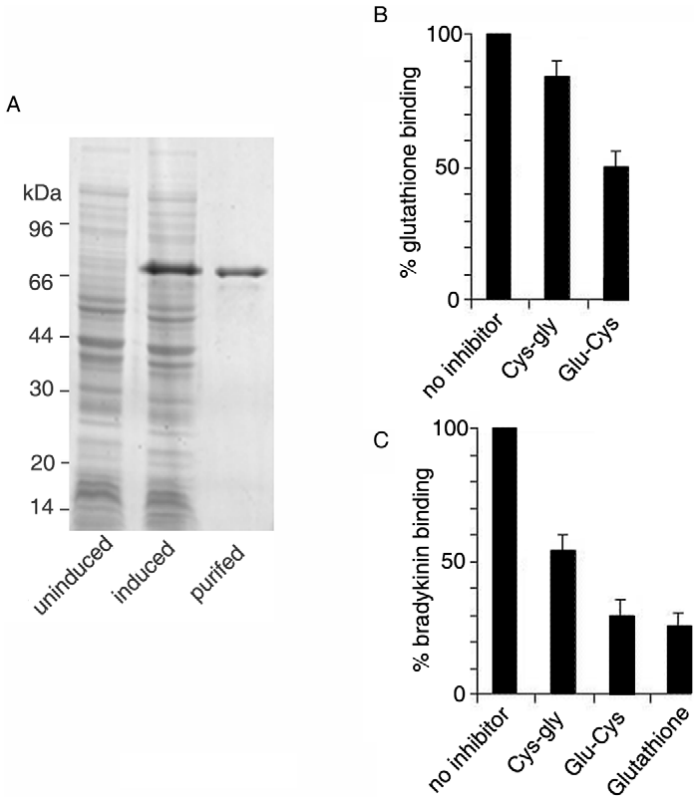
Purification of OppA_MTB_ and its binding with glutathione and bradykinin. (A) Coomassie blue-stained SDS gels showing uninduced, induced *E. coli* lysates expressing His-OppA_MTB_, and purified His-OppA_MTB_. Protein marker sizes are indicated on the left side of the gel. (B,C) In vitro binding assay was carried out with purified OppA_MTB_ using radiolabeled glutathione (B) or bradykinin (C) in the absence (no inhibitor) or presence of unlabeled dipeptide or glutathione (1 mM) as indicated. Data are presented taking the binding in the absence of inhibitor as 100%. Results represent the means ± S.D. of three determinations.

The putative OppA proteins of *M. tuberculosis* and *M. smegmatis* are 68% identical (as analyzed using the BLAST algorithm) [26] (Fig. S1). A putative signal peptide was predicted at residues 34–46 of OppA_MTB_ using the programme Emboss sigcleave (http://emboss.bioinformatics.nl/cgi-bin/emboss/sigcleave). OppA_MTB_ (Gene Bank accession number CAB00902) was expressed and purified as an N-terminal His-tagged protein in *E. coli* BL21(DE3) (Fig. 1A). This recombinant OppA_MTB_ was devoid of the first 56 residues.

The *L. lactis* OppA has been reported to transport peptides from 4 upto 18 residues, including the nonapeptide bradykinin, reflecting its versatility [27]. We tested whether OppA_MTB_ transports the nonapeptide bradykinin (RPPGFSPFR). An in vitro binding assay using radioactive bradykinin at a concentration of 1 mM showed that OppA_MTB_ binds 36±1.5 pmol bradykinin/mg protein (n = 3).

Next, considering a previous report indicating that the Opp transport system is involved in uptake of GSH in *M. bovis* BCG [24], we tested the ability of OppA_MTB_ to bind glutathione. At a concentration of 1 mM GSH, OppA bound 146±1 pmol GSH/mg protein, suggesting that the tripeptide is preferred over the bulkier nonapeptide bradykinin. The GSH-derived dipeptide glu-cys was a more efficient competitor of GSH binding than the peptide cys-gly (Fig. 1B). GSH and its peptides could also competitively inhibit bradykinin binding (Fig. 1C). In summary, the above results show that OppA_MTB_ is capable of binding to peptides of varying size. However, it likely prefers the smaller peptides as substrates.

### Homology modeling and mutational analysis of OppA_MTB_

Sequence similarity searches showed that OppA_MTB_ is 22.1% identical to OppA of *S. typhimurium* and 21% to OppA of *B. subtilis*. The three dimensional structures of OppA proteins are likely more conserved between different species than their primary amino acid sequences. Based on this premise, a three-dimensional model of OppA was built on the basis of fold prediction results. The model encompasses residues I77 to R591. OppA_MTB_ showed three domains selected on the basis of clustering of folds. Domains I and III (Fig. S2) are similar to the corresponding domains in many substrate binding proteins and the interface between domains I and III likely comprises the peptide binding pocket. For the substrate binding proteins of the oligopeptide importers, peptide binding usually involves hydrogen bonding between binding pocket amino acid residues of the protein and the main chain of the ligand [9].

**Figure 2:**
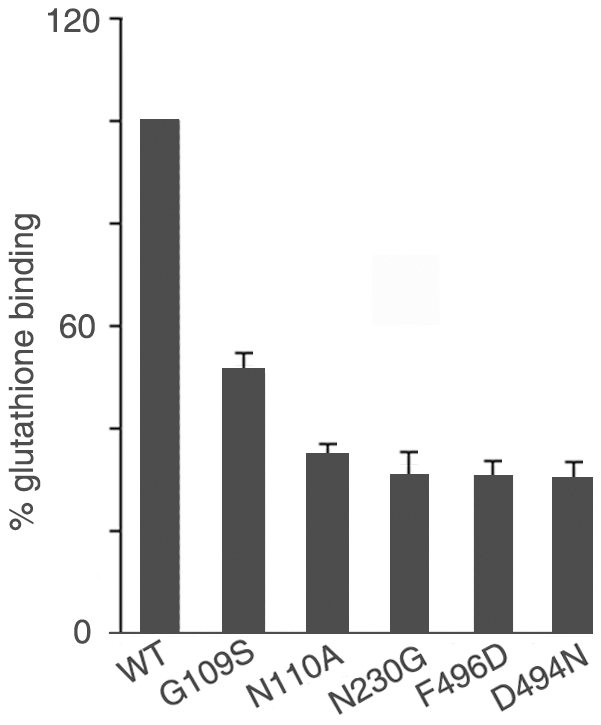
Mutational analysis of glutathione-binding ability of OppA_MTB_. Binding of recombinant wild type (WT) OppA_MTB_ or its mutants to radioactive glutathione was studied as described under “Materials and Methods.” Binding of glutathione to OppA_MTB_ (WT) was set at 100%. Results represent the means ± S.D. of three determinations.

Docking of OppA was carried out with both bradykinin and glutathione as ligands. Docking analyses suggested that the interactions of OppA with ligands involve binding pocket residues containing amide or hydroxyl groups as hydrogen bond donors/acceptors. Amino acids I107, D108, G109, N110, V114, A115, N230, and G231 (present in domain I), S194 and M226 (present in domain II), and W491 and F496 (present in domain III) were predicted to be involved in binding to GSH. Most of these residues were also predicted to be involved in binding of bradykinin. Several of these residues were therefore chosen for mutational analyses. In addition, the residue D494, conserved between *M. tuberculosis* and *B. subtilis* OppA was also chosen for mutation since it is partially exposed in the peptide binding pocket of OppA of *B. subtilis*. The mutations G109S, N110A, N230G, F496D and D494N resulted in more than 50% loss of GSH or bradykinin binding activity (Fig. 2). Mutation N230G affected GSH binding possibly due to the lowering of backbone interaction between the amino acid at position 230 and GSH. Docking analyses suggested that the N-terminal Glu of GSH is important for binding with OppA. This supported our observation that the peptide Glu-cys was a better competitor of GSH for binding to OppA than the peptide Cys-gly.

### Generation of an oppA knock out (OppA-KO) of *M. smegmatis*

**Figure 3:**
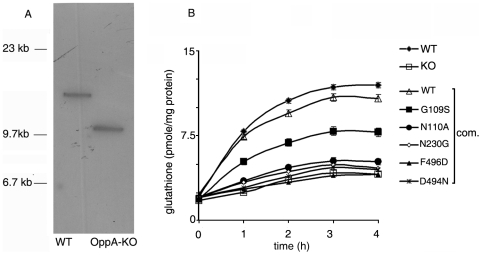
Glutathione uptake in *M. smegmatis.* (A) Southern analysis of wild type (WT) and OppA-KO of *M. smegmatis.* (B) *M. smegmatis* WT, OppA-KO and OppA-KO complemented (com.) with *oppA*_MTB_ [wild type or the indicated mutants] were grown upto logarithmic phase, harvested, washed and incubated with radiolabeled glutathione at 37°C. Aliquots were withdrawn at the indicated time points and uptake was studied by measuring the radioactivity incorporated in the cells (as described under Materials and Methods). Results represent the means ± S.D. of three determinations.

OppA is conserved across both pathogenic and non-pathogenic mycobacterial species suggesting that its function is likely to be conserved. OppA of *M. tuberculosis* and *M. smegmatis* are 81% similar and 68% identical. In view of this, we used the fast-growing nonpathogenic *M. smegmatis* as a model organism to further investigate the effect of mutations of OppA on peptide transport in mycobacteria. Knockout of *oppA* was confirmed by Southern hybridization (Fig. 3A). OppA-KO was complemented with a single copy of His-*oppA*_MTB_ or its mutants by integration into the chromosome of *M. smegmatis* using the integrating vector pOPP206 harboring the His-*oppA_MTB_* gene under the control of the *hsp60* promoter.

### Glutathione uptake in OppA-KO

In order to study the effects of mutations in OppA, the uptake of [^3^H]GSH was measured in OppA-KO and OppA-KO complemented with wild type or mutant versions of *oppA*_MTB_. [^3^H]GSH uptake was lower in OPPA-KO compared to the wild type (Fig. 3B). Complementation with wild type *oppA*_MTB_ restored [^3^H]GSH uptake to levels similar to the wild type. However, complementation with the mutants N110A, N230G, F496D or D494N, did not restore GSH uptake (Fig. 3B). The G109S mutant could partially reverse loss of GSH uptake ability of OppA-KO. These results supported those obtained by in vitro binding assays and also the view that the Opp importer plays a major role in uptake of glutathione by *M. smegmatis*.

### Glutathione uptake in *M. tuberculosis*

**Figure 4:**
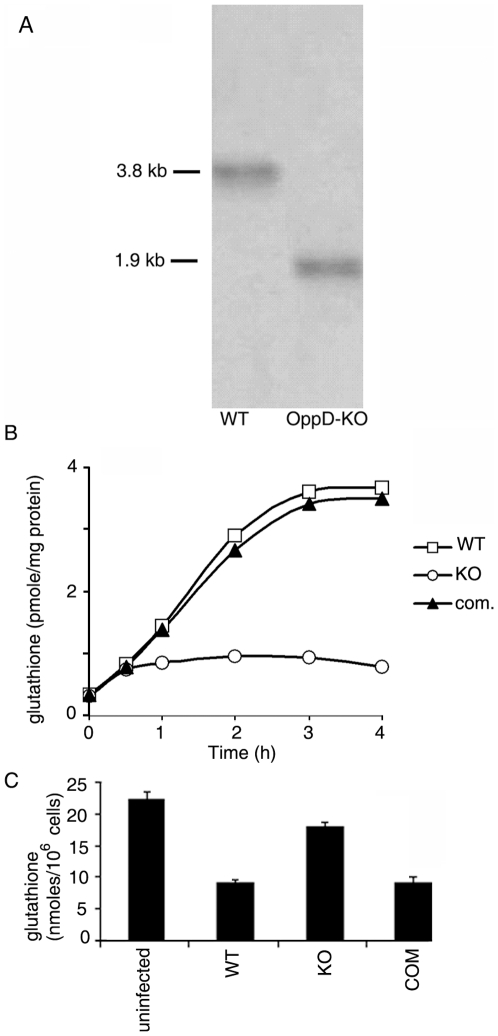
Glutathione uptake in *M. tuberculosis* and glutathione level in infected THP-1 cells. (A) Southern analysis of wild type and OppD-KO of *M. tuberculosis.* (B) *M. tuberculosis* wild type (WT), OppD-KO (KO) or OppD-KO complemented with *oppDA*_MTB_ (com.) was grown upto logarithmic phase, harvested, washed and incubated with radiolabeled glutathione at 37°C. Aliquots were withdrawn at the indicated time points and uptake was studied by measuring the radioactivity incorporated in the cells (as described under “Materials and Methods”). (C) Glutathione pool was measured in uninfected or infected THP-1 macrophages as described under “Materials and Methods”. Results represent the means ± S.D. of three determinations.

The *oppD* gene of *M. tuberculosis*, encoding the two ATP binding components of the transporter, was disrupted by deletion and insertion of a kanamycin resistance cassette and confirmed by Southern hybridization (Fig. 4A). Since *oppA* is located downstream of *oppD*, the insertion of the kanamycin resistance cassette, also blocked expression of *oppA.* An *oppDA*-complemented strain was constructed by inserting a copy of *oppDA* into the chromosome of H37Rv using the integrating vector pOppD109 harboring the *oppDA* genes under the control of the *hsp60* promoter. The growth rates of the knockout and complemented strains were comparable to the wild type in vitro (Fig. S3).

Just as in the case of *M. smegmatis*, uptake of [^3^H}GSH was compromised in the OppDA-KO mutant of *M. tuberculosis* compared to the wild type (Fig. 4B). Uptake was restored upon complementation with *oppDA*. This result supported the view that the Opp transporter of *M. tuberculosis* is also capable of importing GSH. We next tested the hypothesis that GSH uptake by the Opp importer of *M*. *tuberculosis* residing in macrophages may deplete the macrophage GSH pool, by comparing macrophage GSH content after infection by wild type *M. tuberculosis* or the OppD-KO. The GSH pool was significantly depleted in macrophages infected with *M. tuberculosis* compared to uninfected macrophages (Fig. 4C). The extent of depletion of GSH was greatly reduced when macrophages were infected with OppD-KO, whereas the *oppDA*-complemented strain behaved like the wild type. Intracellular GSH levels are significantly reduced in PBMCs from tuberculosis patients compared to normal, healthy controls [28]. Our results suggest that import of GSH by the Opp importer is likely to contribute to this phenomenon.

### Methyl glyoxal (MG) levels in *M. tuberculosis* H37Rv and OppD-KO

**Figure 5:**
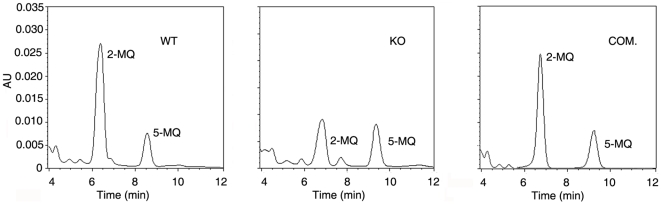
Methlglyoxal (MG) levels in macrophages after infection with *M. tuberculosis.* Differentiated THP-1 cells were infected with *M. tuberculosis* H37Rv (WT), OppD-KO (KO) or OppD-KO complemented with *oppDA*_MTB_ (COM.) at an MOI of 20 for 2 h. Unphagocytosed bacteria were removed, cells were washed, incubated for 24 h in fresh medium, lysed and MG levels were determined in the lysate by HPLC [as 2-methylquinoxaline (2-MQ)] as described under “Materials and Methods” and by Rachman *et al*. [23]. 5MQ was used as an internal standard. The chromatograms are representative of the results obtained from three separate sets of experiments.

During mycobacterial infection of macrophages, the cellular levels of methyl glyoxal (MG) [a physiological product of various metabolic pathways] [29], are elevated. Since GSH is required for the conversion of MG to lactate, we speculated that GSH import by *M. tuberculosis* could impair the ability of the macrophage to detoxify MG. Based on this premise, we assessed MG levels after infection of macrophages (at an MOI of 20) with wild type *M. tuberculosis* H37Rv, OppD-KO and the complemented strains. In three independent experiments, MG levels were 4.8 times higher in wild type *M. tuberculosis*-infected macrophages compared to uninfected macrophages, whereas MG levels were 1.5 times higher (over uninfected macrophages) in OppD-KO- infected cells. MG levels were restored to that observed in macrophages infected with the wild type, when OppD-KO was complemented with *oppDA* of *M*. *tuberculosis* (Fig. 5). These observations suggested that expression of the OppABCD importer of *M*. *tuberculosis* was correlated with MG levels in infected macrophages.

### Macrophages infected with OppD-KO produce lower amounts of TNF-α, IL-1β and IL-6 and undergo reduced apoptosis compared to the wild type

**Figure 6:**
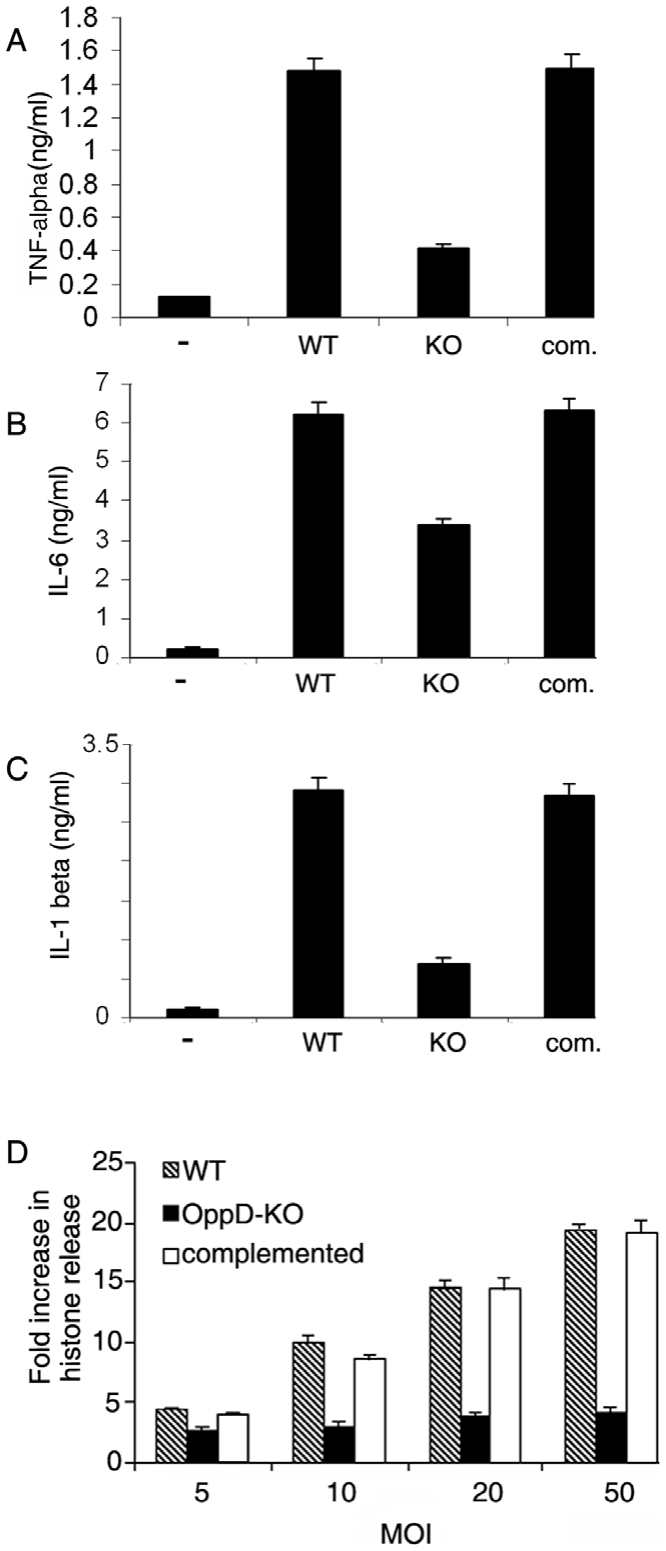
Attenuation of cytokine release and mycobacteria-induced apoptosis in differentiated THP-1 cells by OppD-KO cells. Differentiated THP-1 cells were infected with *M. tuberculosis* H37Rv, the isogenic OppD-KO or OppD-KO complemented with *oppDA* at an MOI of 1:20 as described under “Materials and Methods” (A–C). After 24 h, cytokine release was measured in the supernatants using cytokine assay ELISA kits, or (D) cells were washed, lysed, and cell death was measured using the Cell Death ELISA kit (Roche) as described under “Materials and Methods”. Results of cell death assays are expressed as the fold increase in the release of histone compared to untreated THP-1 cells. Results represent the means ± S.D. of three determinations.

Based on previous reports that MG production is associated with elevated levels of inflammatory cytokine production, we analyzed cytokine/chemokine production in macrophages infected with wild type *M*. *tuberculosis* or OppD-KO at an MOI of 20. At this MOI, cells were treated with bacteria for 2 h, washed, lysed and bacterial load was determined. Comparable CFUs of 1.4×10^4^, 1.5×10^4^ and 1.3×10^4^ bacteria per 10^5^ macrophages were obtained for the wild type, OppD-KO and complemented strains respectively. After 24 h, OppD-KO infection led to lesser amounts of TNF-α, IL-1β and IL-6 production in macrophages compared to the wild type (Fig. 6A-C). These observations were in line with reports that MG levels regulate cytokine production from macrophages. No significant differences were observed in the production of IFN-β, MCP-1, IL-10, IL-8 and RANTES (data not shown).

Since TNF-α is a known proapoptotic cytokine, we tested for observable differences in apoptosis of macrophages infected with *M. tuberculosis* H37Rv or OppD-KO. Apoptosis was lower in macrophages infected with OppD-KO compared to the wild type (Fig. 6D).

## Discussion

Peptide import is likely to be an important process in the biology of the mycobacteria as for other bacteria. *Streptomyces coelicolor* and *Corynebacterium glutamicum*, two species belonging to the same taxonomic order as mycobacteria, have often provided important clues regarding functions of gene products in the pathogen *M. tuberculosis*. In *Streptomyces*, the oligopeptide transport system, Opp imports an oligopeptide that is likely involved in regulating development [30]. Signaling molecules that likely regulate processes such as entry into and exit out of dormancy in mycobacteria, remain to be identified. Whether the peptide importers play any role in developmental processes in mycobacteria, remains open to question. As a step in this direction, we undertook characterization of one of the putative peptide transporters of *M. tuberculosis*, annotated as an ABC transporter *oppABCD*, where *oppA* encodes the substrate binding lipoprotein. In this study we confirm that OppA is a peptide-binding protein. We present evidence that OppA binds to a wide range of peptides in terms of their size, ranging from the nonapeptide bradykinin to the tripeptide GSH. It shows a preference for binding glutathione compared to bradykinin, In addition, we demonstrate that GSH-derived dipeptides can compete for peptide binding, suggesting that the substrate specificity extends to dipeptides as well. Our results differ somewhat from those of Flores-Valdez *et al*. (2009) who have argued that OppABCD is a dipeptide transporter. Using homology modeling to predict amino acid residues of the peptide-binding pocket of OppA, followed by mutational analysis, we demonstrate that amino acid residues G109, N110, N230, D494 and F496 are involved in peptide binding. Microarray expression profiling has clearly shown that the *Rv3665c-Rv3663c* locus in *M. tuberculosis* regulates at least some genes which are induced during nutrient deprivation and hypoxia [24]. It would be of considerable interest to analyze the transcriptome of the OppD-KO strain described by us under different growth conditions.

Most importantly, we report the fortuitous observation of a novel role of OppABCD in modulating the outcome of *M. tuberculosis*-macrophage interactions. Increase in the macrophage MG levels (over the level in uninfected macrophages) is 4.8 fold in the case of macrophages infected with the wild type vs. 1.5 fold in the case of macrophages infected with OppD-KO. TNF-α stimulates the production of reactive oxygen intermediates (ROI) [31]. TNF-α production by *M. tuberculosis* could therefore impair GSH-redox status by production of ROI. Our studies suggest that in addition to the aforesaid mechanism, other mechanisms exist for reduction of GSH levels in infected macrophages. Glutathione uptake by mycobacteria through the *opp* system could be one such mechanism. The O'Connell laboratory has shown that GSH is toxic to “in-vitro grown” *M. bovis* BCG [13]. However, our studies pertain to *M*. *tuberculosis* H37Rv rather than to *M. bovis* BCG. The O'Connell laboratory has shown hypersurvival of the *dpp* mutant, suggesting that this could be due to the impaired ability of the mutant to import glutathione. It could just as well be contended that the differences in CFU occur because of attenuated apoptosis of macrophages infected with the *dpp* mutant. Death of macrophages would rob the bacterium of its niche for replication, therefore accounting for differences in CFU between the two strains.

Apoptosis of macrophages in granulomas in TB patients, is induced by an environment rich in MG. Rachman *et al*. [23] have shown that pretreatment with GSH leads to significant reduction in mycobacteria-induced apoptosis. Glutathione uptake by mycobacteria through the *opp* system could therefore contribute towards enhanced apoptosis of infected macrophages. Mycobacteria-induced apoptosis was studied by infecting differentiated THP-1 cells with wild type *M*. *tuberculosis*, OppD*-*KO, and the *oppDA*-complemented strain. There was marked reduction of apoptosis of macrophages infected with OppD-KO compared with the wild type and restoration of the apoptosis-inducing ability in the complemented strain. We contend that the reduced glutathione uptake by OppD-KO increases GSH levels inside the macrophages compared to cells infected with the wild type strain, thereby facilitating detoxification of MG. As a result, apoptosis in OppD-KO-infected macrophages is reduced in comparison with macrophages infected with the wild type *M. tuberculosis*. MG is associated with the induction of inflammatory cytokines such as TNF-α [32]. Our results suggest that import of GSH by the OppABCD importer, and subsequent impairment of MG-detoxification in infected macrophages, contributes to enhanced production of inflammatory cytokines such as TNF-α, IL-6 and IL-1β. This is partly rescued in OppD-KO. We contend that these are not non-specific effects due to differences in CFUs for wild type and OppD-KO grown in macrophages, since the observations are restricted to a subset of cytokines.

Apoptosis of host macrophages is required for established an effective immune response against tuberculosis [33]. Our observations therefore suggest that it would be of interest to compare the ability of the *oppD* mutant to cause disease and maintain bacterial burden during both the chronic and the persistent phases of infection.

## Supporting Information

Table S1 Primers for amplification of derivatives of OppA of *M. tuberculosis*.
(0.01 MB PDF)

Figure S1 Blast analysis of OppA of *M. tuberculosis* and *M. smegmatis*.
(0.01 MB PDF)

Figure S2 Stereoview of the model of *M. tuberculosis* OppA.
(0.21 MB PDF)

Figure S3 Growth curves of wild type *M. tuberculosis*, OppD-KO and oppDA-complemented OppD-KO.
(0.04 MB PDF)
