# Supplementary material for: An Oligopeptide Transporter of Mycobacterium tuberculosis Regulates Cytokine Release and Apoptosis of Infected Macrophages
Source: PLoS One. 2010 Aug 17;5(8):e12225. doi: 10.1371/journal.pone.0012225 (PMC2923189; doi:10.1371/journal.pone.0012225)
Supplement: Table S1 — Primers for amplification of derivatives of OppA of M. tuberculosis. (0.01 MB PDF) [file pone.0012225.s001.pdf]

Table S1.

Primers for amplification of derivatives of $OppA_{MTB}$

Primers for OppA derivatives

Primer b	Primer c	Mutation
5’- AACATCTTGCACGACGACGGCAAC-3’	5’-GTTGCCGTCGTCGTGCAAGATGTT -3’	I 107S
5’-TTGCACATCGCCGGCAACAAC-3’	5’-GTTGTTGCCGGCGATGTGCAA-3’	D108A
5’-TTGCAATCGACAGCAACAACGCC -3’	5’-GGCGTTGTTGCTGTCGATGTGCAA -3’	G109S
5’-ATCGACGGCGCCAACGCCGAG-3’	5’-CTCGGCGTTGGCGCCGTCGAT-3’	N110A
5’-AACAACGCCGAGGACGCGGCGATG-3’	5’-CATCGCCGCGTCCTCGGCGTTGTT –3’	V114D
5’- AACGCCGAGGTCGAGGCGATGATG-3’	5’-CATCATCGCCTCGACCTCGGCGTT -3’	A115D
5’-GAGTGGCGCGGTAAGTTCGCGGGC-3’	5’-GCCCGCGAACTTACCGCGCCACTC-3’	M226N
5’-TTCGCGGGCAACTACATGCTGCTG-3’	5’-CAGCAGCATGTAGTTGCCCGCGAA -3’	G231Y
5’-ATCGCTTCTGGCAGCGGCGCC-3’	5’-GGCGCCGCTGCCAGAAGCGAT-3’	S194G
5’-GCCCAATACGCCCTCACCAGC-3’	5’-GCTGGTGAGGGCGTAAACCCG-3’	G377R
5’-GACATCGCAGCGTTCGGCTGG-3’	5’-CCAGCCGAACGCTGCGATGTC-3’	Q488A
5’-GCACAGTTCGGCTCGGTGGGCGAC -3’	5’-GTCGCCCACCGAGCCGAACTGTGC -3’	W491S
5’-TGGGTGGGCAACGCGTTTCCG-3’	5’-CGGAAACGCGTTGCCCACCCA-3’	D494N
5’-GTGGGCGACGCGGTTCCGCTGTCA-3’	5’- TGACAGCGGAACCGCGTCGCCAC-3’	F496D

“a” 5’-TTTCTAGA**CATATG**GCTGACCGTGGCCAG-3’ (NdeI site in bold)

“d” 5’-TAT**AAGCTT**CAGCGTCGCATGAACCC-3’ (HindIII site in bold)
