# Supplementary material for: An Oligopeptide Transporter of Mycobacterium tuberculosis Regulates Cytokine Release and Apoptosis of Infected Macrophages
Source: PLoS One. 2010 Aug 17;5(8):e12225. doi: 10.1371/journal.pone.0012225 (PMC2923189; doi:10.1371/journal.pone.0012225)
Supplement: Figure S1 — Blast analysis of OppA of M. tuberculosis and M. smegmatis. (0.01 MB PDF) [file pone.0012225.s002.pdf]

```
Mtb    45   VAMVLTGCSASGTQLELAPTADRRAAVGTTSDINQQDPATLQDGGNLRLSLTDFPPNFNI  104
            V M +T C++    +   P+A   A VG T+DIN QD A L+ GGNLRL+L  FP NFN
Msmeg  24   VTMAMTSCTSDERDI---PSAGGTAEVGATNDINPQDVANLRQGGNLRLALPGFPSNFNT  80

Mtb    105  LHIDGNNAEVAAMMKATLPRAFIIGPDGSTTVDTNYFTSIELTRTAPQVVTYTINPEAVW  164
            LHIDGN A +  +M+ATLPRA+ +GPDGSTTV+T+YFTS+ELT T PQVVTYTINPEAVW
Msmeg  81   LHIDGNEASLGGLMRATLPRAYRVGPDGSTTVNTDYFTSVELTSTDPQVVTYTINPEAVW  140

Mtb    165  SDGTPITWRDIASQIHAISGADKAFEIASSSGAERVASVTRGVDDRQAVVTFAKPYAEWR  224
            +DGTPITW DIA+QIHA SG DKAF+IAS +G++RVASVTRGVDDRQAVVTFA+P++EW+
Msmeg  141  TDGTPITWEDIAAQIHATSGKDKAFQIASLNGSDRVASVTRGVDDRQAVVTFAEPFSEWQ  200

Mtb 225     GMFAGNGMLLPASMTATPEAFNKGQLDGPGPSAGPFVVSALDRTAQRIVLTRNPRWWGAR  284
            GMFAGN MLLP S T+ PE FNKGQL+GPGPSAGPF+VS +DR AQRI LTRNP+WWG
Msmeg  201  GMFAGNTMLLPKSSTSDPEVFNKGQLNGPGPSAGPFMVSNVDRGAQRITLTRNPKWWGEP  260

Mtb    285  PRLDSITYLVLDDAARLPALQNNTIDATGVGTLDQLTIAARTKGISIRRAPGPSWYHFTL  344
            P LDSITY VLDDAAR+PALQNN +DATGV +LD+L IA RT GISIRRAP PSWYHFT
Msmeg  261  PVLDSITYTVLDDAARIPALQNNALDATGVASLDELEIARRTPGISIRRAPSPSWYHFTF  320

Mtb    345  NGAPGSILADKALRLAIAKGIDRYTIARVAQYGLTSDPVPLNNHVFVAGQDGYQDNSGVV  404
            NGAPGSILADKALR A+AKGIDR  I  VAQ GL  DP PLNNH+FVAGQ+GYQDNS VV
Msmeg  321  NGAPGSILADKALRQAVAKGIDRNIITTVAQRGLADDPKPLNNHIFVAGQEGYQDNSEVV  380

Mtb    405  AYNPEQAKRELDALGWR-RSGA-FREKDGRQLVIRDLFYDAQSTRQFAQIAQHTLAQIGV  462
            A++PE+AK ELDALGW  + GA FREKDG++L IRD+ YD+QSTRQ A IAQ++LAQIGV
Msmeg  381  AFDPEKAKAELDALGWTLKPGAQFREKDGKRLTIRDVLYDSQSTRQIATIAQNSLAQIGV  440

Mtb    463  KLELQAKSGSGFFSDYVNVGAFDIAQFGWVGDAFPLSSLTQIYASDGESNFGKIGSPQID  522
            +L ++AK GSGFF+D++  G FDIAQF WVGDAF L  L QIY +D +SNFG+I SP++D
Msmeg  441  ELRIEAKPGSGFFTDWIIPGNFDIAQFSWVGDAFSLCCLNQIYTTDADSNFGRISSPEVD  500

Mtb    523  AAIERTLAELDPGKARALANQVDELIWAEGFSLPLTQSPGTVAVRSTLANFGATGLADLD  582
                 T+ ELDP KAR +AN++D+LIW E FSLPL QSPG VAVRS LAN+G  G+ D+D
Msmeg  501  TKAIATMGELDPEKARTMANELDKLIWEEVFSLPLFQSPGNVAVRSNLANYGPAGIGDID  560

Mtb    583  YTAIGFMR  590
             T +GFM+
Msmeg  561  ITKVGFMK  568
```
