# Supplementary material for: An Oligopeptide Transporter of Mycobacterium tuberculosis Regulates Cytokine Release and Apoptosis of Infected Macrophages
Source: PLoS One. 2010 Aug 17;5(8):e12225. doi: 10.1371/journal.pone.0012225 (PMC2923189; doi:10.1371/journal.pone.0012225)
Supplement: Figure S2 — Stereoview of the model of M. tuberculosis OppA. (0.21 MB PDF) [file pone.0012225.s003.pdf]

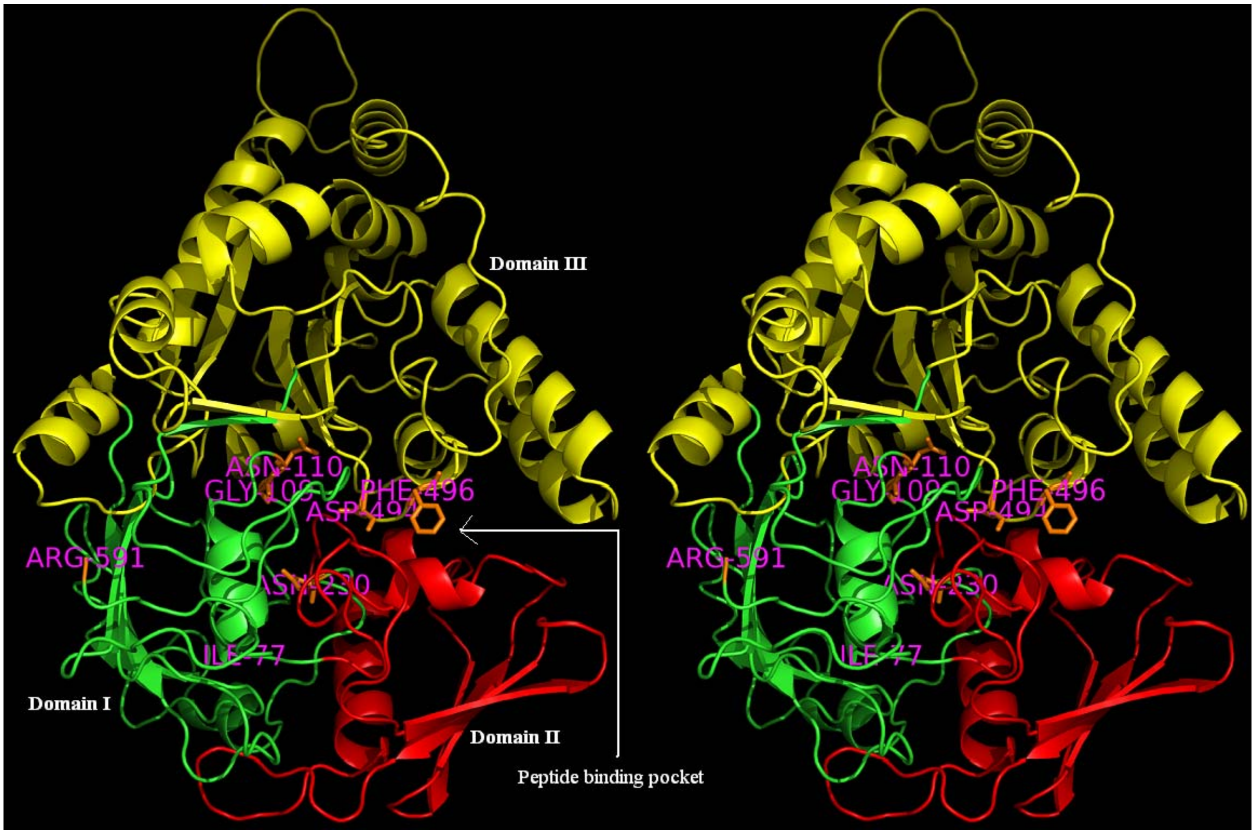

Amino acid residues I77 to R591 of $OppA_{MTB}$ was modeled as described in Materials and Methods. The three domains are shown in different colors: domain I (green), domain II (red), and domain III (yellow). The peptide-binding pocket resides in the interface between domains I and III. The residues of OppA *viz*., G109, N110, N230, F496 and D494 that were mutated resulting in more than 50% loss of GSH and bradykinin activity have been highlighted.
