# Supplementary figures and images for: An Oligopeptide Transporter of Mycobacterium tuberculosis Regulates Cytokine Release and Apoptosis of Infected Macrophages
Source: PLoS One. 2010 Aug 17;5(8):e12225. doi: 10.1371/journal.pone.0012225 (PMC2923189; doi:10.1371/journal.pone.0012225)

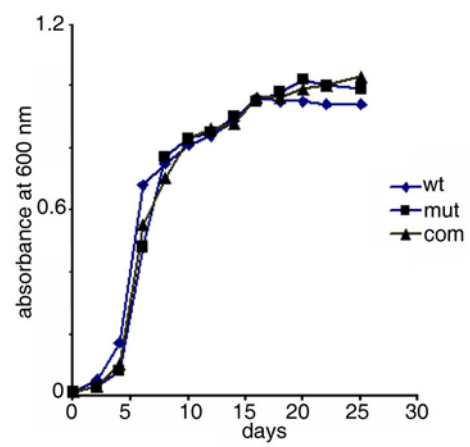

Supplement: Figure S3 — Growth curves of wild type M. tuberculosis, OppD-KO and oppDA-complemented OppD-KO. (0.04 MB PDF) [file pone.0012225.s004.pdf]
